# Potential Risk Factors for the Development of Self-Injurious Behavior among Infants at Risk for Autism Spectrum Disorder

**DOI:** 10.1007/s10803-017-3057-9

**Published:** 2017-02-20

**Authors:** Adele F. Dimian, Kelly N. Botteron, Stephen R. Dager, Jed T. Elison, Annette M. Estes, John R. Pruett, Robert T. Schultz, Lonnie Zwaigenbaum, Joseph Piven, Jason J. Wolff, The IBIS Network

**Affiliations:** 1 Department of Educational Psychology, University of Minnesota, 56 East River Rd., Minneapolis, MN 55455 USA; 2 Department of Psychiatry, Washington University in St. Louis, St. Louis, MO USA; 3 Department of Radiology, University of Washington, Seattle, WA USA; 4 Institute of Child Development, University of Minnesota, Minneapolis, MN USA; 5 Department of Speech and Hearing Sciences, University of Washington, Seattle, WA USA; 6 Center for Autism Research, Children’s Hospital of Philadelphia, Philadelphia, PA USA; 7 Department of Pediatrics, University of Alberta, Edmonton, AB Canada; 8 Department of Psychiatry, University of North Carolina at Chapel Hill, Chapel Hill, NC USA

**Keywords:** Self-injurious behavior, Repetitive behavior, Autism spectrum disorder, Risk factors, Infants

## Abstract

Prevalence of self-injurious behavior (SIB) is as high as 50% among children with autism spectrum disorder (ASD). Identification of risk factors for the development of SIB is critical to early intervention and prevention. However, there is little empirical research utilizing a prospective design to identify early risk factors for SIB. The purpose of this study was to evaluate behavioral characteristics predicting SIB at age 2 years among 235 infants at high familial risk for ASD. Logistic regression results indicated that presence of SIB or proto-SIB and lower developmental functioning at age 12 months significantly predicted SIB at 24 months. A pattern of persistent SIB over this period was associated with a diagnosis of autism and poorer cognitive and adaptive outcomes.

## Introduction

Repetitive behavior is a core diagnostic feature of autism spectrum disorder (ASD). Self-injurious behavior (SIB) is a form of repetitive motor behavior that is both self-directed and has the potential to result in tissue damage (Lewis and Bodfish 1998; Tate and Baroff 1966). Examples of SIB topographies include head hitting, head banging, skin picking and pinching, hair pulling, and self-biting (Rojahn et al. 2008). SIB point-prevalence estimates (i.e., the number of cases of SIB at one point in time divided by the number of persons in a defined population at the same point in time) vary widely depending on age and diagnosis, but estimates range from 2 to 24% in community/total population studies of individuals with intellectual disabilities (Cooper et al. 2009). For individuals with autism spectrum disorder (ASD), point-prevalence estimates for SIB have been reported to be as high as 53% among children (Baghdadli et al. 2003; Duerden et al. 2012), and 69% among adults with the disorder (Bodfish et al. 2000). While more rigorous epidemiological studies are needed, existing work suggests that SIB is a relatively common behavior disorder that occurs across the lifespan of individuals with ASD. The deleterious effects associated with SIB, such as risk of permanent injury and interference with the acquisition of adaptive behaviors, can negatively impact the quality of life of affected individuals and their families (e.g., Emerson 1990; Emerson et al. 2001b; Eyman and Call 1977; Symons and Thompson 1997; Taylor et al. 2011).

There are currently no established prevention programs targeted specifically at reducing the incidence of SIB among children with neurodevelopmental disorders such as ASD. Although there have been promising findings from a limited number of prevention oriented studies utilizing functional communication training (e.g., Fahmie et al. 2016; Luszynski and Hanley 2013; Reeve and Carr 2000; Richman 2008), there is a need to first clarify the clinically-relevant risk factors for the development and persistence of SIB. The extant literature on putative risk factors and associated variables with SIB varies greatly in terms of the methodology, measurement tools, and the target populations and ages investigated (Furniss and Biswas 2012; MacLean et al. 2010; McClintock et al. 2003; Rojahn et al. 2008). The number of observational studies to date specific to SIB is impressive; their findings, however, can be difficult to generalize across diagnostic categories (e.g., etiologically defined disorders, ASD, idiopathic intellectual disability, and at-risk/ developmental delay groups) and age groups in particular (McClintock et al. 2003).

Previous research on adults with a diagnosis of ASD suggests that SIB may be more prevalent (Richards et al. 2012) and of greater severity (Bodfish et al. 2000) in comparison to individuals with intellectual disabilities. When it comes to identified risk factors (i.e., a factor that directly increases the probability of SIB occurring and is part of a causal chain) and risk markers (i.e., an attribute that is associated with increased probability of SIB, but is not necessarily causal), there are disparate findings and mostly data on the latter (Burt 2001). In a meta-analysis conducted by McClintock et al. (2003), common risk markers for SIB among various samples of children and adults with intellectual disabilities included an autism diagnosis, severity of autism, level of intellectual functioning, communication deficits, and the presence of certain syndromic neurodevelopmental disorders (e.g., Lesch-Nyhan syndrome). Among^2008^ the few longitudinal studies of individuals with intellectual disabilities, the three most reported risk markers for the persistence of SIB in adolescents and adults are lower receptive and expressive language (Chadwick et al. 2008; Emerson et al. 2001a; Kiernan and Alborz 1996; Nøttestad and Linaker 2001; Schroeder et al. 1978), lower daily living skills and adaptive behavior (Chadwick et al. 2008; Emerson et al. 2001a; Kiernan and Alborz 1996; Nøttestad and Linaker 2001), and intellectual disability (Cooper et al. 2009; Nøttestad and Linaker 2001; Schroeder et al. 1978).

The potential risk factors and markers specific to the early development of SIB, however, have been examined primarily cross-sectionally and retrospectively (e.g., Fodstad et al. 2012). Berkson et al. (2001) were among the first to follow a group of young children with developmental disabilities who were receiving birth to three early intervention services (3–40 months old). The group reported that onset of SIB occurred on average at age 16 months. Similarly, Kurtz et al. (2003) and Richman and Lindauer (2005) reported that both SIB and proto-injurious SIB (proto-SIB; topographies similar to SIB that do not cause tissue damage) emerge before or at 25 months of age. Proto-SIB has been identified as a potential risk marker for the emergence of SIB (Furniss and Biswas 2012; Petty et al. 2009; Richman and Lindauer 2005; Symons et al. 2005). Repetitive rhythmic motor stereotypies, such as body rocking and hand flapping, have also been considered as a potential behavioral precursor to SIB (Baumeister and Forehand 1973; Rojahn et al. 2015). Through extended contact with the social environment, certain motor stereotypies may be shaped into topographies of SIB and possibly become sensitive to social reinforcement (Guess and Carr 1991; Kennedy 2002; Oliver et al. 2005). Empirical studies addressing this model of SIB development are limited and findings overall have been mixed (Furniss and Biswas 2012). Causal relationship aside, there is evidence that motor stereotypy is associated with SIB (Barnard-Brak et al. 2015; Oliver et al. 2012; Petty et al. 2009; Rojahn et al. 2012) and may predict its occurrence (Barnard-Brak et al. 2015; Richman et al. 2012; Rojahn et al. 2015).

Prospective, longitudinal cohort designs are essential for identifying predictive temporal relations and provide stronger evidence for causal inferences than retrospective cohort and cross-sectional designs (Aschengrau and Seage 2014). Only two sets of prospective cohort studies and one direct observation study have investigated early SIB among young children at risk for developmental delay (Rojahn et al. 2015; Schroeder et al. 2014) and with developmental disabilities (Berkson 2002; Berkson et al. 2001; Richman and Lindauer 2005) that included some children with ASD. With a sample of young children at risk for a behavior disorder such as aggression or SIB from Peru (n = 180; age = 4–48 months), Schroeder et al. (2014) examined potential risk factors across three time points. Results indicated that SIB varied by diagnostic group status over time. More specifically, children screening positive for ASD engaged in high rates of SIB at time 1 that decreased over time, while children with Down syndrome showed low levels of SIB at time 1 that increased modestly thereafter. Using the same data from Schroeder et al. (2014) and Rojahn et al. (2015) examined the relationship of motor stereotypy to SIB over time using latent growth modeling. The authors concluded that the best fitting model included stereotypy as a predictor of later SIB. Berkson (2002) examined age trends among young children receiving early intervention services, finding that SIB emerged early on, with certain topographies, such as head banging, appearing first. Richman and Lindauer (2005) also examined emerging SIB over time among young children with developmental delay (n = 12; age = 14–32 months) using functional analyses. Each analysis was individualized and stereotypy, proto-SIB and SIB were targeted and followed over time. The results indicated that the topography and function of the target behavior stayed the same for most of the participants. While most participants in that study showed both motor stereotypies and proto-SIB at study entry, the latter behavior changed over time to include new topographies or to increase in severity (causing tissue damage) for 5 of 12 children. Taken together, SIB onset patterns appear to vary dynamically over time in relation to diagnostic status and to other forms of repetitive behavior.

To date, there are no prospective cohort studies of SIB among young children at high familial risk for ASD, who are defined as such by virtue of having an older sibling with the disorder, during the first years of life. Because SIB is an early-emerging behavioral disorder associated with autism and developmental delay (Dominick et al. 2007; Duerden et al. 2012), the focus of the current study was to downward extend the literature on potential risk factors for SIB development and persistence to infants at high risk for ASD. Specifically, we examined cognitive and behavioral characteristics at age 12 months in relation to presence or absence of SIB at age 24 months in a longitudinal study of 235 children at familial high risk for ASD.

## Methods

### Participants

Study participants were from the Infant Brain Imagining Study (IBIS), an ongoing longitudinal multisite study of infants at high familial risk for ASD. Participants were recruited from across the United States through research registries, flyers, brochures, community clinics, websites, and email blasts. Assessments were performed at one of four clinical data collection sites including Children’s Hospital of Philadelphia, University of North Carolina, University of Washington, and Washington University in St. Louis. Exclusion criteria entailed: (1) evidence of a specific genetic condition or syndrome; (2) significant medical or neurological condition affecting development; (3) significant vision or hearing impairment; (4) birth weight <2000 g or gestational age < 36 weeks; (5) significant perinatal adversity or prenatal exposure to neurotoxins, (6) contraindication for MRI, (7) predominant home language other than English, (8) children who were adopted or half siblings, (9) 1st degree relative with psychosis, schizophrenia, or bipolar disorder, and (10) twins. Familial high-risk status was defined by having an older sibling with a community diagnosis of the ASD confirmed by the Autism Diagnostic Interview-Revised (ADI-R; Lord et al. 1994) and Social Communication Questionnaire (SCQ; Rutter et al. 2003).

The present study included a sample of infant siblings considered to be at high risk for ASD, for whom cognitive and behavioral assessment batteries were completed at 12 and 24 months of age (n = 235). The cognitive and behavioral assessments included a parent-report measure of repetitive and self-injurious behavior. For the purpose of providing context, descriptive data on SIB is also provided for a sample of low-risk control infants; however, this group was not included in subsequent analyses given our study aims and overall low base rate of SIB in this group [n = 95; SIB at 24 months = 14/95 (14.7%)]. Low-risk infants were recruited and assessed as part of the parent study. Low-risk infants met the exclusion criteria described above and had typically developing older siblings as confirmed by the SCQ and no first-degree relatives with ASD or intellectual disability. High- and low-risk infants had complete Repetitive Behavior Scales-Revised (RBS-R) at both 12 and 24 months. Subgrouping of high-risk participants on the basis of diagnostic status at age 2 was based on clinical best-estimate using DSM-IV-TR criteria made by experienced, licensed clinicians using all available clinical and developmental assessment data, with confirmation by a second senior clinician blind to risk and diagnostic status. Study procedures were approved by institutional review at each clinical assessment site with informed consent documented for all participants.

### Measures

*The Repetitive Behavior Scales—Revised* (RBS-R; Bodfish et al. 2000) is a parent or caregiver rated measure of restricted and repetitive behaviors comprised of 43 discrete behavioral topographies. The RBS-R provides scores for total repetitive behavior as well as for six subtypes thereof. RBS-R measures of interest to the present study were inventories of self-injurious behavior (SIB) and stereotypical motor. The SIB subscale was the primary dependent variable and was used as a binary grouping variable (SIB or no SIB) based on the presence or absence of any SIB item endorsed by caregivers at 24 months. The SIB and stereotypical motor subscales were also used to catalogue the number and type of these behaviors at ages 12 and 24 months. The RBS-R, including our subscales of interest, captures individual differences in behavior among toddlers at high-risk for ASD (Wolff et al. 2014) and has been independently validated for use in young children (Mirenda et al. 2010).

*The Autism Diagnostic Observation Schedule* (ADOS; Lord et al. 2000) is a semi-structured diagnostic assessment designed to probe for symptoms associated with ASD. The ADOS was used to generate a standardized symptom severity score (Gotham et al. 2009) as well as domain scores for repetitive behavior and social affective symptoms. Assessment data from the ADOS also contributed to the determination of diagnostic classification. While updates have been made to the ADOS subsequent to the initiation of our longitudinal study, use of the ADOS-G was maintained to ensure consistency across subjects and time.

*The Mullen Scales of Early Learning* (MSEL; Mullen 1995) is a standardized developmental assessment designed for children ages 0–68 months. The Early Learning Composite (ELC) score, an index of overall cognitive and behavioral development, was used to provide an estimate of overall developmental quotient. The ELC is a standard score with *M* = 100, *SD* = 15. Subscales from the MSEL were used in secondary analyses and included expressive and receptive language, fine and gross motor, and visual reception. These subscales yield T-scores with *M* = 50, *SD* = 10. Separate non-verbal and verbal developmental quotients were also calculated based on age-equivalent scores.

*Vineland Adaptive Behavior Scales-II* The Vineland (Sparrow et al. 2005) is a standardized and norm-referenced assessment of adaptive function based on a semi-structured parent interview. The Vineland provides an Adaptive Behavior Composite (ABC) score as well as indexes of adaptive function in each of four subdomains, including socialization, which indexes interpersonal relationships, play and leisure skills, and interpersonal coping skills. This subdomain was of specific interest given a previously observed inverse relationship between socialization skill and repetitive behavior in toddlers who developed ASD (Wolff et al. 2014). Although daily living skills have been previously linked to SIB in older individuals (Kiernan and Alborz 1996; Emerson et al. 2001b; Nøttestad and Linaker 2001; Chadwick et al. 2008), this Vineland subdomain was not examined given questionable relevance to toddlerhood. The Vineland ABC and Socialization scores are standardized, *M* = 100, *SD* = 15.

### Statistical Analyses

To explore the relationship between the variables of interest and the outcome (presence or absence of SIB as defined by the RBS-R), descriptive and correlational analyses were conducted to characterize the high risk infants included in this study. Point-prevalence (number of participants with SIB, new and preexisting, at one time point divided by the total sample), cumulative incidence (number of participants who developed SIB in the specific time period divided by the number of participants at risk of developing SIB at the beginning of the period), and relative risk (cumulative incidence in the exposed group, i.e. group with the potential risk factor, divided by the cumulative incidence in the unexposed group) estimates were calculated (Aschengrau and Seage 2014). Next, a series of logistic regression models were fitted to test which psychosocial variables (based on the extant literature) at 12 months (time 1) were predictive of parent-endorsed SIB (SIB or no SIB) at 24 months (time 2). Odds ratios were calculated based on these models for each predictor variable tested. Bootstrap sampling with replacement (B = 1000) was used to generate confidence interval estimates. Finally, secondary analyses using one-way ANOVA were conducted to further characterize and explore possible group-level differences in select cognitive and behavioral features between participants based on SIB status at times 1 and 2 (persistent SIB, incident SIB, transient SIB, and no SIB). This included MSEL composite and subscale scores and Vineland adaptive composite and socialization score. Post-hoc pairwise comparisons were performed following the omnibus ANOVA and corrected using the Tukey method.

## Results

The primary study sample of high risk infants included 62.6% males and was 87.3% white. The mean age at 12 and 24 months assessment dates were 12.5 (*SD* = 0.6) and 24.8 (*SD* = 1.5) months, respectively (see Table 1 for descriptive and demographic information). Of the 235 high risk infants, 47 (20%) met clinical best-estimate criteria for ASD at age 24 months (combined autistic disorder or pervasive developmental disorder, not otherwise specified).

**Table 1:** Descriptive and demographic characteristics

Demographics/variables	Total sample (n = 235)
Males (%)	62.6%
Race/Ethnicity	
White/ Caucasian	87.3%
Black	5.1%
Asian	0.6%
More than one race/ethnicity	7.0%
Maternal education	
No college degree	30.4%
College degree	36.7%
Graduate degree	32.3%
Household Income	
<$50k	21.5%
$50k–$100k	34.8%
>$100k	38.0%
Not answered	5.7%
Mean age at 12 month assessment	12.55 (0.62)
Mean age at 24 month assessment	24.83 (1.47)
MSEL early learning composite at 12 months	98.53 (13.69)
Vineland adaptive behavior composite at 12 months	95.66 (9.85)
ASD diagnosis at 24 months	19.9%

*MSEL* Mullen scales of early learning; standard scores are reported for the MSEL and Vineland (*M* = 100)

### Prevalence

At age 12 months, the point prevalence estimate for children who engaged in SIB was approximately 39%. In comparison, at age 24 months, the point prevalence estimate for children who exhibited SIB was 32%. Of the children whose parents endorsed at least one topography of SIB at 12 months, 48% persisted in engaging in SIB at 24 months.

### Incidence and Persistence

At 24 months of age, there were 31 incident cases of SIB (i.e., new cases of SIB at 24 months). The cumulative incidence estimate (i.e., absolute risk) was 0.22 over 12 months. Among the children who engaged in SIB at both 12 and 24 months, 47% decreased in the total number of topographies of SIB (i.e., total SIB items endorsed), while 28% were reported to have an increase in number of topographies. The remaining 26% of children who persisted in SIB from 12 to 24 months were reported to have the same overall number of topographies of behavior at both time points. For low risk infants, cumulative incidence was 0.08 and persistence of SIB from 12 to 24 months was approximately 6%. Cumulative incidence and persistence were significantly lower for low risk infants in comparison to high-risk infants (Fisher’s exact test; *p* < .001 and *p* = .003, respectively).

### Relative Risk

One or more topographies of SIB were endorsed at either time point for 74% of participants with an ASD diagnosis and 47% for those without. A relative risk estimate was calculated comparing participants who received a diagnosis at 24 months compared to those who did not. The results indicated that the risk of engaging in SIB at 24 months was 1.85 times higher among children who were later diagnosed with ASD compared to children who did not receive a diagnosis.

### ASD Diagnosis by SIB Group

A chi-square test was performed to evaluate if a diagnosis of ASD significantly differed by SIB status. The four groups compared were children who engaged in SIB at both 12 and 24 months (persistent n = 44), children who started engaging in SIB at 24 months (incident n = 31), children who engaged in SIB at 12 months but not at follow up (transient n = 48), and children who had no SIB at either time point (no SIB n = 112). There was a significant relationship between a diagnostic group status and SIB status, *X* ^2^ (3, n = 235) = 10.1, *p* = .02. Children meeting criteria for ASD at age 24 months constituted 32% of the persistent group, 36% of the incident group, 19% of the transient group, and 14% of the no SIB group.

### SIB Subscale Items

**Fig. 1:**
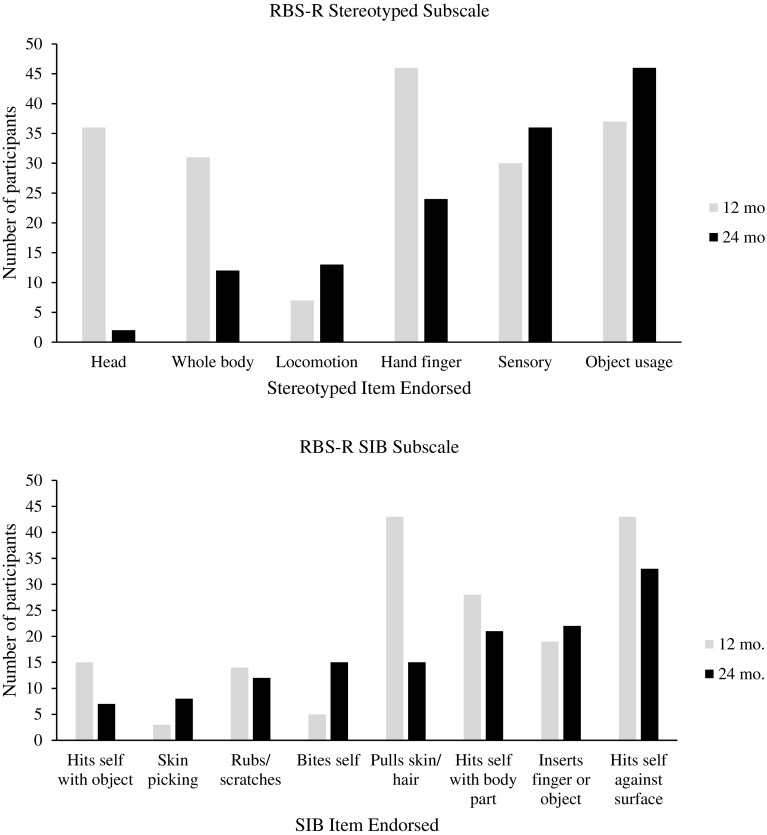
Caregiver endorsement of items on RBS-R stereotyped and self-injurious behavior subscales

At 12 months, participants engaged in hitting against a surface and pulling skin or hair the most (Fig. 1). The least endorsed item within the SIB subscale at 12 months was skin picking. Caregivers also endorsed hitting self against a surface the most at 24 months. The least endorsed item at 24 months was hits self with an object. SIB only increased in terms of item endorsement for bites self and inserts finger or object. Overall, all other forms decreased at 24 months, with pulling hair and/or skin decreasing the most.

**Fig. 2:**
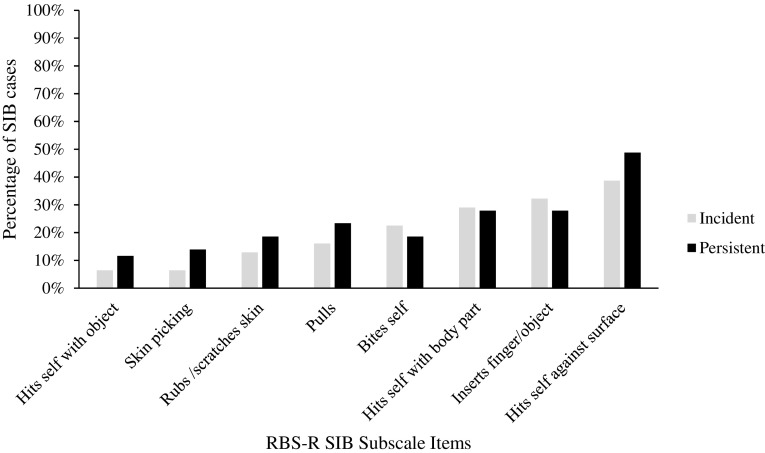
Self-injurious behavior items endorsed by caregivers at 24 months among incident cases (n = 31) and persistent cases (n = 43)

The SIB items endorsed were also compared at 24 months between participants who engaged in SIB at both time points (i.e., persistent cases) and those who just started engaging in SIB at Time 2 (i.e., incident cases) (Fig. 2). A similar pattern was observed at 24 months with the both the incident and persistent SIB cases engaging in hits body against a surface the most, with 39 and 49%, respectively. The least endorsed item for both types of cases was hits self with object, (7% incident and 12% persistent). Overall, proportions of reported topographies on the SIB subscale were relatively similar between incident and persistent cases.

### Stereotyped Behavior Subscale Items

Figure 1 also displays the topographies endorsed (item-level RBS-R data) for stereotyped behavior on the RBS-R. At 12 months, caregivers endorsed hand finger stereotyped behavior the most, and locomotion the least. At 24 months for the stereotyped behavior subscale, caregivers endorsed repetitive object usage the most, and repetitive head movement the least. Overall, topographies of stereotyped behaviors that decreased from 12 to 24 months at assessment time included head, whole body, and hand finger. Conversely, topographies that increased in terms of caregiver endorsement at 24 months were locomotion, sensory, and object usage.

### Risk Factors

Four logistic regression models (Table 2) were fitted to examine which characteristics significantly predicted SIB at 24 months among all high-risk infants. The first model included all variables of interest, based on the literature, including sex, MSEL ELC (developmental quotient), Vineland ABC (adaptive behavior), and endorsed SIB and stereotyped behavior items from the RBS-R. Of these predictors, only MSEL ELC score and SIB at 12 months were statistically significant predictors of SIB at 24 months at (*X* ^2^ = 35.83, df = 5, p < 0.001). Subsequent to this result, Model B was fit with MSEL ELC, endorsed items of SIB, and endorsed items of stereotyped behavior at age 12 months. In Model B, again only MSEL ELC and SIB endorsement were statistically significant predictors. For Model C, we tested only MSEL ELC and stereotyped behavior in relation to later SIB. Both factors were statistically significant. For a final Model D, we tested the contribution of MSEL ELC and SIB items endorsed. Both predictors were statistically significant and model fit was similar to that of Models A and B and superior to Model C. Overall, the results of logistic regression analysis indicated that for participants who exhibited SIB at 12 months, the odds of engaging in SIB at 24 months was between 75–92%. Consistent across models, the odds of SIB at Time 2 decreased by 3% for each unit increase in the MSEL ELC score. Goodness-of-fit was relatively consistent across Models A, B, and D, with R^2^_pseudo_ = 0.21 for A and B, and R^2^_pseudo_ = 0.19 for Model D. Goodness-of-fit for Model C, which included MSEL ELC and stereotyped behavior, was less robust with R^2^_pseudo_ = 0.13.

**Table 2:** Unadjusted odds ratios with 95% confidence intervals between psychosocial characteristics at 12 months and SIB at 24 months

Predictor	Model A OR [CI]	Model B OR [CI]	Model C OR [CI]	Model D OR [CI]
MSEL ELC	0.97* [0.94,0.99]	0.97** [0.94, 0.99]	0.97** [0.95, 0.99]	0.97* [0.94,0.99]
RBS-R SIB endorsed	1.75*** [1.26,1.91]	1.75*** [1.27, 1.90]	–	1.92*** [1.45,2.54]
RBS-R stereotyped endorsed	1.18 [0.92,1.45]	1.20 [0.94, 1.45]	1.42** [1.17,1.61]	
Vineland adaptive behavior composite	0.99 [0.95,1.02]	–	–	–
Sex	1.1 [0.75,1.43]	–	–	–
*Omnibus X* ^2^	35.8***	36.8***	22.4***	34.3***
*Nagelkerke R* ^2^	0.21	0.21	0.13	0.19

*MSEL ELC* mullen early learning composite, *RBS-R* repetitive behavior scale, revised

*p < 0.05, **p < 0.01, ***p < 0.001

### Secondary Analyses

Table 3 displays the results of a one-way ANOVA to test mean differences in MSEL ELC and subscale scores and select Vineland scores between children who persisted in engaging in SIB from 12 to 24 months of age, those who did not (i.e., no SIB or transient), and incident cases of SIB at 24 months. There were statistically significant mean differences between groups for the MSEL composite score as well as receptive language, gross motor, and visual reception subscales (see Table 3). For the Vineland, there were statistically significant mean differences between groups for both adaptive composite score and socialization scores.

**Table 3:** Mean differences between SIB groups on MSEL and Vineland-II at age 24 months

	Persistent (n = 44)		Incident (n = 31)		Transient (n = 48)		No SIB (n = 112)		*F*	*p*
	M	SD	M	SD	M	SD	M	SD		
MSEL										
Composite (ELC)	90.3^a^	18.3	92.4^ab^	20.6	100.3^b^	17.9	100.6^b^	16.7	4.7	0.003
Expressive language	43.4	11.4	43.4	11.4	48.5	13.2	47.5	11.1	2.5	0.06
Receptive language	43.4^a^	13.5	44.7^ab^	44.7	50.1^ab^	12.3	50.3^b^	12.2	3.6	0.02
Fine motor	46.7	12.0	45.2	11.9	48.2	8.8	49.3	9.8	1.6	0.20
Gross motor	44.8^a^	9.3	48.0^a^	9.9	50.4^b^	11.2	48.0^ab^	8.7	3.7	0.01
Visual reception	45.6^a^	9.7	49.4^ab^	13.4	53.3^b^	10.4	53.2^b^	10.9	5.8	0.001
Vineland-II										
Composite	93.3^a^	10.2	96.8^ab^	10.8	100.8^b^	12.0	99.9^b^	8.7	5.4	0.001
Socialization	93.4^a^	10.9	96.9^ab^	11.7	100.6^b^	13.0	100.5^b^	9.0	5.2	0.002

P value is omnibus comparison. Post-Hoc Tukey HSD test results with mean differences indicated by letters a and b. Means that do not share a letter are significantly different (*p* < .05); means that share a letter are not significantly different. *MSEL* mullen scales of early learning, *SIB* self-injurious behavior. MSEL ELC and Vineland-II Composite and Socialization scores are standardized (*M* = 100); MSEL subscale scores at T-scores

Post-hoc analyses corrected for multiple comparisons suggested that groups with no or transient SIB (i.e., SIB at 12 months but not at 24 months) did not differ significantly from one another and were characterized by higher scores across MSEL and Vineland measures relative to the persistent and incident SIB groups. Children in the persistent SIB group had the lowest MSEL and Vineland scores overall, and their scores were significantly lower than those of the transient and no-SIB groups on the majority of MSEL and Vineland measures. The incident SIB group was intermediate to the persistent and transient SIB groups.

## Discussion

The purpose of this study was to evaluate characteristics at age 12 months that predicted self-injurious behavior (SIB) at age 24 months among infants at familial high risk for ASD. We were particularly interested in downward extending findings from the extant literature on SIB in order to test putative risk markers for later emerging SIB. In the most parsimonious model, we found that SIB at 12 months in an infant’s repertoire and lower developmental/intellectual functioning significantly predicted the emergence and/or persistence of SIB at age 24 months among infants at high risk. Contrary to some of the extant literature on potential risk markers (e.g. Emerson et al. 2001b; Rojahn et al. 2015), we did not find strong evidence for motor stereotypy as a predictor of SIB. Indeed, stereotypy was only modestly predictive of later SIB in a model which did not account for early manifestations of SIB-related topographies. This may be due to how highly correlated SIB and stereotyped behavior are or may indicate that specific topographies of stereotypy, versus stereotypy in general, are associated with SIB. For example, in a cross-sectional sample of 1871 children and adults with intellectual disabilities, Barnard-Brak et al. (2015) found that stereotyped behavior was a strong predictor of SIB for 69% of participants but not for the remaining 31%. Their results also indicated that specific topographies of stereotypy (yelling and body rocking) may predict specific forms of SIB, versus a more general relationship between these classes of behavior. Alternatively, the present findings may indicate that the relationship between stereotypy and SIB qualitatively differs for children at-risk for ASD during early development.

With regard to the relationship of SIB to stereotypy, there are two issues which merit consideration. First, there is evidence that, despite some topographical similarity, SIB and stereotypy may be distinct phenomenon in terms of both behavior (Bishop et al. 2013; Mirenda et al. 2010; Richler et al. 2007; Wolff et al. 2016) and underlying neurobiology (Wolff et al. 2013). Second, as opposed to stereotypies in general, it is feasible that the SIB topographies reported (i.e., endorsed SIB items) among our sample of toddlers at risk for ASD more closely reflect proto-SIB as originally conceived (Berkson et al. 2001; Richman and Lindauer 2005). That is, stereotyped motor behaviors which have the *potential* to cause tissue damage (e.g., light head or leg slapping, banging of objects against self, or hand mouthing) but that have not yet risen to a pivotal level of severity or concern (e.g., audible self-directed hitting that produces red marks or bruises; hand mouthing that results in chapped hands or other tissue damage).

While our sample overall was relatively typically developing as indicated by mean cognitive and adaptive behavior scores, a substantial minority will be characterized by atypical development in the form of ASD or a related neurodevelopmental or psychiatric disorder by school age (Miller et al. 2016). At age 24 months, approximately 20% of high-risk children met diagnostic criteria for ASD. SIB occurred at a higher rate among children receiving a diagnosis of ASD, but was not exclusive to this subset of high-risk infants. The relative risk of a child with ASD engaging in SIB at 24 months was almost two-fold that of a child without a diagnosis. These data are consistent with previous work and suggest that SIB emerges early in life, can be persistent, and is prevalent among children with ASD (e.g., Baghdadli et al. 2008; Berkson et al. 2001; Schroeder et al. 2014). Almost half of the participants who engaged in SIB at Time 1 persisted in engaging in SIB at follow up. Other studies report high persistence estimates among individuals with intellectual disabilities (Taylor et al. 2011) and children with PDD-NOS (Baghdadli et al. 2008). Based on these data, it is likely that once proto-injurious behavior or SIB emerges, it may remain stable, and should be evaluated even if it is not yet severe yet.

A strength of this study was its use of a longitudinal and prospective cohort design, adding to a very limited published literature using such an approach to the study SIB among young children with or at-risk for a developmental disability. With the prospective cohort design, we were able to calculate the cumulative incidence of SIB (i.e., new cases of SIB that developed over a period of time) over a 12-month period. There are very few cumulative incidence estimates of SIB reported in the literature. Incidence estimates are needed for the assessment of prevention trials (i.e., the impact of the program on the incidence of SIB) and so the inclusion of this estimate may lay the groundwork for future research. Berkson et al. (2001) estimated that among 39 children under the age of 40 months receiving early intervention services for general developmental delay, incident cases of SIB were 1.3% over 1–3 years of follow up. Murphy et al. (1999) followed an older sample of 614 children with intellectual disabilities (under the age of 10 years) and reported a cumulative incidence of 3%. Among a sample of adults with intellectual disabilities, Cooper et al. (2009) reported a cumulative incidence of SIB of 0.6% over 2 years of follow up (n = 651). Measurement variability and sample characteristics likely contribute to this range of cumulative incidence estimates. Results from the present study pertain to a particular risk group over a focused age interval: toddlers at familial high risk for autism from 12 to 24 months of age. While we expect that our findings may not generalize to other risk groups or ages, they do provide specific targets for further study, including the possibility of developing early intervention or prevention strategies.

In general, the developmental progression of early SIB is not well understood. Results from two published cross-sectional studies and one longitudinal study suggest that head banging is the most common early SIB topography (Berkson et al. 2001; MacLean and Dornbush 2012; Kurtz 2012). Hand biting and hand mouthing are also common SIB topographies reported for young children with intellectual and developmental disabilities (Murphy et al. 1999; Hall et al. 2001; Richman and Lindauer 2005). Within the present sample, changes in SIB topographies (endorsed items on the SIB subscale) were observed from 12 to 24 months. Hitting self against surface (e.g., head banging) was the most commonly endorsed topography at 12 and 24 months, and tended to remain in the children’s repertoires across time points and across SIB groups (incident, persistent, and transient). Overall, a majority of the items endorsed (47%) decreased from ages 12 to 24 months. For example, skin and hair pulling decreased sharply over this interval. Other forms showed an increase, as with self-biting. An early developmental pattern of decreasing SIB, function notwithstanding, has been reported in typically developing children among whom such behaviors are relatively common and often associated with tantrums (Berkson and Tupa 2000; Hoch et al. 2015). It may be that stability of SIB into toddlerhood is a clinically relevant feature that merits specific consideration in future prospective research (Emerson et al. 2001a). Given that those with a diagnosis of ASD were almost twice as likely to engage in SIB at 24 months than those who did not receive a diagnosis, it may be prudent to monitor early repetitive behavior in general and SIB in particular closely during the first years of life for children who are at high risk.

In an attempt to further elucidate risk markers predicting SIB within our sample, secondary analyses focused on MSEL subscales and the Vineland were performed. We examined group-level differences in cognitive and behavioral features among children based on whether their SIB from 12 to 24 months was persistent, transient, or incident, as well as those with no reported SIB. The results of these analyses suggest that those with persistent and, to a lesser extent, incident SIB were characterized by lower cognitive and adaptive behavior scores relative groups with transient or no SIB. Effects were strongest for measures of receptive language, gross motor skill, visual reception, and adaptive and socialization skills. These differences are consistent with correlational evidence in the SIB literature (e.g., Matson et al. 2009) and may inform early intervention strategies targeted to skill acquisition in specific functional domains, such as receptive language or social and play skills, as inoculation against SIB risk.

## Limitations

The present study relied on parent reported repetitive behavior, aggregating SIB into two categories (i.e., SIB or no SIB at 24 months based on parent endorsement of any SIB item on the RBS-R). Direct observation of SIB would preclude potential biases associated with proxy report of behavior and could also provide information about the frequency or function of SIB (e.g., Richman and Lindauer 2005). Further, our analyses relied on reports of occurrence of SIB without regard to severity. This limitation is related in part to use of the RBS-R, which is a clinical measure not necessarily suited to detecting severity among very young children. Indeed, it is not clear how a parent would judge severity of self-directed behaviors performed by an infant or toddler. Future work might address this issue through more developmentally appropriate or objective means of quantifying severity, perhaps clarifying the distinction and developmental relationship between SIB and proto-SIB. Because the sample evaluated was not restricted to only incident SIB cases in the logistic regression analyses, we were unable to directly assess which predictors preceded the emergence of SIB or proto-SIB. Optimally, recruitment of a cohort of infants at high risk for ASD that are not engaging in any SIB at study entry, and prior to the age of 12 months, would facilitate research specific to the emergence of SIB, while following children to later ages would provide a clearer understanding as to the natural progression of SIB and SIB-like behavior. While we did not detect a significant effect for sex in predicting SIB, this does not preclude the possibility that such effects were masked by a disproportionately male sample.

In closing, the purpose of this study was to evaluate cognitive and behavioral characteristics predicting early SIB among children at high familial risk for ASD. With only a handful of studies utilizing a prospective research design to identify risk factors for SIB during the first years of life, this study provides an initial examination of risk factors associated with SIB among young children who are at elevated risk for ASD. Continued efforts to ameliorate the deleterious effects and high treatment costs associated with SIB are warranted, and one promising strategy is to pursue preventative approaches by identifying early risk factors.
